# Artificial intelligence-assisted speaking in Spanish as a foreign language: effects on foreign language emotions and willingness to communicate

**DOI:** 10.3389/fpsyg.2026.1877956

**Published:** 2026-08-27

**Authors:** Yifan Miao, Pingping Hu

**Affiliations:** 1 School of International Communication, Institute of International Studies, Wenzhou Business College, Wenzhou, China; 2 School of Foreign Languages, Wenzhou University of Technology, Wenzhou, China

**Keywords:** foreign language emotions, language other than English, longitudinal study, Spanish speaking, willingness to communicate

## Abstract

**Introduction:**

Although AI-powered conversational tools are increasingly used in foreign language education, little is known about their longitudinal effects on learners’ emotional experiences and willingness to communicate (WTC), particularly among beginner learners of languages other than English. Grounded in Positive Psychology, Control-Value Theory, and the WTC framework, this study examined the effects of AI-assisted speaking practice using TalkPal AI on foreign language enjoyment (FLE), foreign language anxiety (FLA), foreign language boredom (FLB), and WTC in Spanish as a foreign language.

**Methods:**

This longitudinal quasi-experimental study involved 69 Chinese university students with no formal prior learning experience in Spanish, who were assigned to an experimental group (*n* = 35) and a control group (*n* = 34) based on intact classes. Quantitative data were collected at three time points using validated questionnaires. Semi-structured interviews with nine purposively selected students from the experimental group were analyzed thematically to contextualize the quantitative findings. Quantitative data were analyzed using linear mixed-effects models, planned contrasts, and change-score correlation analyses.

**Results:**

No significant short-term group differences were observed during the early stage of the intervention. Across the full intervention period, the experimental group showed a significantly greater reduction in FLB than the control group. At the final measurement point, the experimental group reported significantly higher WTC and significantly lower FLB, while FLE showed a positive but non-significant tendency and no significant group difference was observed for FLA. In the experimental group, increases in FLE were positively associated with increases in WTC over time, whereas changes in FLB and FLA were not significantly related to WTC. Qualitative findings further showed that learners gradually reported greater enjoyment and engagement and less monotony as AI-mediated interaction became routinized over time. Learners also perceived AI-supported speaking practice as a psychologically safe rehearsal space, although evaluation-related anxiety and the limited emotional authenticity of AI interaction remained salient.

**Discussion:**

These findings suggest that AI-assisted conversational practice may exert delayed and differentiated effects on beginner learners’ emotional experiences and willingness to communicate, primarily by reducing boredom and supporting WTC rather than alleviating anxiety.

## 1 Introduction

Emotions occupy a central place in second language acquisition (SLA) research (Prior, 2019), with growing recognition that affective experiences profoundly shape how learners engage with, persist in, and ultimately succeed in language learning (Jiang and Dewaele, 2023; Teimouri et al., 2022). With the introduction of Positive Psychology (PP) into SLA, increasing attention has been paid to both positive and negative emotions, moving beyond a deficit-focused view of affect toward a more comprehensive understanding of learners’ emotional lives (MacIntyre and Mercer, 2014; Dewaele and MacIntyre, 2014). Within this framework, foreign language enjoyment (FLE), foreign language anxiety (FLA), and foreign language boredom (FLB) have been conceptualized as a dynamic “affective triad” representing distinct yet interacting dimensions of learners’ emotional experience (Li et al., 2023). These three emotions capture positive, negative activating, and negative deactivating dimensions of classroom affect that are particularly relevant to sustained speaking practice, whereas emotions such as pride and shame are more closely tied to specific achievement-related or self-evaluative episodes. Rather than functioning as simple opposites, these emotions may co-occur and interact in complex ways, jointly shaping various aspects of learners’ language learning (Dewaele and Pavelescu, 2019; Feng et al., 2023).

Despite the growing body of research on language learners’ emotions, most existing studies have adopted cross-sectional or short-term designs, capturing only a snapshot of learners’ affective states at one moment (Zhang et al., 2025). Given the inherently dynamic and context-sensitive nature of emotions (MacIntyre and Gregersen, 2012; Dewaele and Li, 2020), there remains a lack of longitudinal evidence on how multiple emotions co-develop over time, particularly in classroom-based environments.

Beyond their internal dynamics, these emotions carry important consequences for learners’ behavioral engagement in the classroom, particularly their willingness to communicate (WTC) in the target language (Khajavy et al., 2018). WTC is widely regarded as a central goal of foreign language education (MacIntyre et al., 1998), particularly in speaking-oriented courses where oral interaction is essential for language development (Chen, 2023). Learners’ readiness to communicate is not solely determined by linguistic competence, it is also strongly shaped by their emotional experiences (Dewaele and Dewaele, 2018). Empirical findings regarding the relationships between different emotions and WTC, however, remain mixed, particularly with respect to boredom, which has shown inconsistent predictive effects across studies (Li et al., 2022; Kruk, 2022; Alrabai, 2022).

At the same time, the rapid development of artificial intelligence (AI) has created new opportunities for language learning. AI-mediated speaking environments, such as conversational agents, can provide low-pressure, on-demand interactional opportunities, thereby enabling repeated and self-paced oral practice without immediate evaluation (Huang et al., 2022; Tai and Chen, 2023). Such environments may reduce affective barriers, enhance learners’ sense of control, and foster more positive emotional experiences, thereby facilitating communication. Although previous studies have shown that technology-enhanced learning can reduce anxiety and promote enjoyment (Reinders and Wattana, 2014; Lee and Hsieh, 2019), empirical evidence regarding how AI-mediated interaction shapes learners’ emotional trajectories and consequently their WTC over time remains limited.

Furthermore, existing research has been overwhelmingly conducted in English learning contexts, with comparatively little attention to languages other than English (LOTE). This imbalance matters because LOTE learners may show affective patterns that do not simply mirror those found in EFL settings; for example, Dewaele and MacIntyre (2022) found that LOTE learners reported slightly higher FLE and flow, lower FLCA, and stronger links among these affective variables than EFL learners. In the Chinese context, Spanish is typically learned as a second foreign language at the university level, where learners often have limited opportunities for authentic interaction outside the classroom (Wen and Clément, 2003). This restricted interactional ecology may intensify affective experiences while making classroom-based speaking practice particularly influential for learners’ willingness to communicate. However, how AI-assisted learning influences emotional development and WTC in such LOTE contexts remains underexplored.

To address these gaps, the present study examines how AI-assisted speaking practice shapes the longitudinal development of FL emotions (FLE, FLA and FLB) and WTC among Chinese university learners of Spanish, and how emotional changes dynamically relate to communicative readiness over the course of an instructional intervention.

## 2 Literature review

### 2.1 Emotions in foreign language learning

#### 2.1.1 Foreign language anxiety

A substantial body of empirical research has consistently documented the predominantly debilitating role of FLA in foreign language learning. Meta-analytical and large-scale studies have demonstrated a robust negative association between FLA and language achievement, suggesting that higher anxiety is generally linked to poorer performance outcomes (Botes et al., 2020; Teimouri et al., 2019; Zhang, 2019). In speaking-oriented contexts specifically, FLA may be particularly salient given the immediacy of oral production and the heightened risk of public evaluation, making speaking tasks a high-stakes affective environment where anxiety can substantially impede communicative participation (Woodrow, 2006).

Research on FLA in AI-mediated speaking environments has begun to attract attention, though evidence remains nascent. Studies suggest that conversational agents and AI-assisted speaking tools may reduce learners’ anxiety by lowering the evaluative stakes associated with peer and teacher observation (Lee and Hsieh, 2019; Jeon et al., 2023). However, AI-mediated contexts may also introduce technology-specific anxiety, as learners may feel uncertain about learning to use, understand, or interact with AI systems (Wang and Wang, 2022). Zhang et al. (2024) found that technology-enhanced speaking practice significantly reduced FLA among Chinese EFL learners over a six-week intervention. Nevertheless, longitudinal evidence examining how FLA trajectories unfold under sustained AI-assisted oral practice, particularly in LOTE contexts such as Spanish, remains limited.

#### 2.1.2 Foreign language enjoyment

Recent research has emphasized the developmental and dynamic nature of FLE. At early stages, FLE is often experienced as a situation-specific and task-bound emotional state that fluctuates in response to immediate instructional conditions (Boudreau et al., 2018). This situational sensitivity suggests that FLE is closely linked to classroom interactional ecology and therefore particularly responsive to pedagogical interventions. With sustained engagement, enjoyment may gradually shift toward the trait end of the affective continuum, functioning as a more stable affective disposition that may persist across extended periods (Li and Dewaele, 2024). Such state–trait transformation implies that repeated positive classroom experiences may accumulate over time, consolidating transient enjoyment into a more enduring affective orientation toward language learning. This perspective highlights the importance of examining FLE not only as an immediate classroom emotion, but also as a changeable affective experience that may develop across repeated learning episodes.

Longitudinal studies have shown that enjoyment is more sensitive to instructional practices and contextual variation than anxiety, displaying greater responsiveness to changes in the learning environment (Pan and Zhang, 2021; Dewaele et al., 2023b). This relative malleability positions FLE as a particularly promising target for instructional innovation, as shifts in classroom structure, interactional patterns, and task design may translate more readily into measurable affective change.

Moreover, empirical research has consistently demonstrated that FLE should not be conceptualized merely as the positive opposite of FLA. Dewaele and MacIntyre (2014) reported only a weak negative correlation between enjoyment and anxiety in large-scale survey data, a finding later confirmed by meta-analytic evidence (Botes et al., 2022). This partial independence indicates that enjoyment and anxiety constitute related yet distinct emotional systems rather than opposite poles of a single continuum. Consequently, learners may simultaneously experience enjoyment and anxiety during language learning, underscoring the importance of examining multiple emotional dimensions within the same instructional context.

In AI-mediated learning environments, FLE may be particularly amenable to enhancement. The low-pressure, self-paced nature of AI conversational interaction reduces evaluative threat and allows learners to focus on communicative achievement rather than accuracy, potentially broadening the positive emotional experiences associated with speaking practice (Tai, 2026). Relatedly, Fan and Zhang. (2024) found that FLE mediated the relationship between learners’ attitudes toward AI-assisted learning and their continued intention to engage in AI-assisted EFL learning, further highlighting the role of enjoyment in sustaining AI-supported language learning. However, whether AI-assisted oral practice can support sustained changes in FLE across repeated learning episodes, especially among beginner LOTE learners with limited authentic speaking opportunities outside the classroom, has not been empirically examined. The present study approaches this issue by tracing FLE change patterns across three time points, while treating trait-level transformation as a theoretical possibility rather than a directly tested outcome.

#### 2.1.3 Foreign language boredom

FLB in the foreign language classroom is typically manifested as an unpleasant emotional experience marked by disengagement, inattention, dissatisfaction, impatience, and a diminished sense of meaning or goal-directedness in learning activities (Kruk and Zawodniak, 2018; Li et al., 2023).

Empirical research has identified a wide range of learner-internal and learner-external factors contributing to the experience of FLB. Learner-internal factors include control and value appraisals, two core antecedents identified in Control-Value Theory, as well as achievement goals, language proficiency, and physical state (Li and Dewaele, 2020), whereas learner-external factors encompass task difficulty, teaching style, classroom climate, teacher personality, and class organization (Derakhshan et al., 2021; Kruk, 2022; Pawlak et al., 2020). This dual-layered structure suggests that boredom is jointly shaped by cognitive appraisal mechanisms and immediate instructional ecology, rendering it highly context-contingent.

FLB shows considerable variability across tasks, classroom contexts, and teaching modalities, reflecting its strong contextual sensitivity to instructional conditions (Dewaele et al., 2024). Longitudinal research has revealed that higher levels of boredom are associated with lower language achievement and proficiency, although the directionality of this relationship may be asymmetrical, with achievement predicting subsequent boredom more strongly than boredom predicting later achievement (Li et al., 2024). These findings highlight the pedagogical significance of monitoring and addressing boredom in classroom settings, particularly given its strong sensitivity to instructional conditions.

Despite this sensitivity to instructional conditions, empirical research on FLB in AI-assisted speaking contexts has received minimal attention (Zhang and Liu, 2025). AI conversational environments may alleviate boredom by offering task novelty, varied interactional input, and learner-controlled pacing, factors that address common antecedents of boredom identified in classroom research (Jalambo et al., 2025). However, this boredom-reducing effect may not be linear. Given that boredom in language instruction has been closely associated with repetitiveness, monotony, and predictability (Kruk et al., 2021), the novelty of AI interaction may initially counteract monotony and increase situational interest, but boredom may re-emerge if AI-mediated exchanges become predictable, repetitive, or insufficiently challenging over time. Yet whether AI interaction can sustain reduced boredom over an extended instructional period, and whether FLB trajectories in LOTE speaking contexts mirror those documented in English-dominated studies, remains an open empirical question. The present study directly addresses this gap by tracking FLB longitudinally across an AI-assisted Spanish speaking course.

Research on FL emotions has expanded considerably in scope and methodology over the past decade. Studies have examined FLE, FLA, and FLB across a range of instructional contexts, including general classroom settings (Dewaele et al., 2023a), writing tasks (Wu and Halim, 2024), and technology-mediated learning environments (Dong et al., 2026). The majority of this work has focused on English as a foreign language (EFL) contexts (Resnik et al., 2025), with comparatively limited attention to LOTE such as Spanish, where distinct interactional ecologies and learner trajectories may produce different affective dynamics (Dewaele and MacIntyre, 2022). Methodologically, cross-sectional survey designs have predominated, capturing affective states at a single time point and leaving the longitudinal co-development of multiple emotions underexplored (Dewaele and Li, 2020), particularly in classroom intervention settings.

### 2.2 Willingness to communicate in foreign language learning

Willingness to communicate (WTC) was originally conceptualized in L1 communication as a relatively stable personality trait reflecting individuals’ general predisposition to initiate communication (McCroskey and Richmond, 1987). In foreign language contexts, where communication is characterized by greater uncertainty and affective risk, WTC was reconceptualized as a situation-dependent construct and defined as “a readiness to enter into discourse at a particular time with a specific person or persons, using an L2” (MacIntyre et al., 1998, p. 547). Consistent with this situational definition, the present study focuses on classroom-based WTC as learners’ context-sensitive readiness to speak Spanish during instructional activities, rather than as a stable trait-like predisposition.

To account for this increased complexity, MacIntyre et al. (1998) proposed a six-layered pyramid model of L2 WTC, distinguishing between situational antecedents and more enduring individual and social factors. At the situational level, WTC is directly shaped by state communicative self-confidence and the desire to communicate with a specific interlocutor. More distal layers involve motivational, affective-cognitive, and broader social-contextual influences that indirectly shape learners’ readiness to communicate. This model is particularly relevant to AI-mediated speaking practice because AI may alter two immediate situational antecedents of WTC: the conversational partner and the learner’s desire to communicate. As an always-available and nonjudgmental interlocutor, AI can reduce the interpersonal risk of initiating speech, while contingent prompts, follow-up questions, and topic continuity may provide learners with more immediate reasons to continue speaking.

Building on this model, subsequent empirical research on WTC has evolved through trait-oriented, situation-specific, and dynamic-contextual perspectives. Early studies have identified several relatively stable individual factors shaping learners’ WTC, including personality traits (MacIntyre and Charos, 1996), self-confidence in L2 ability (Aoyama and Takahashi, 2020; Khajavy et al., 2018), motivation (Ducker, 2022; Lee and Lu, 2023), and anxiety (Pavelescu, 2023; Wang et al., 2022). In addition to individual factors, contextual conditions also play a crucial role; variables like the interlocutor (Cao, 2014), topic (Pawlak et al., 2016), classroom dynamics (Lee and Drajati, 2019), and task requirements (Tai and Chen, 2023) can substantially influence learners’ WTC.

Research has increasingly emphasized the situational and dynamic nature of WTC, highlighting its variability across time and learning contexts. Longitudinal and case-based studies have shown that learners’ WTC can change as a function of developing linguistic competence, increased self-confidence, reduced foreign language anxiety, and growing familiarity with classroom procedures and interactional norms (de Saint Léger and Storch, 2009; Cao, 2013; Mystkowska-Wiertelak, 2021). These findings position WTC not as a fixed disposition, but as a context-sensitive readiness state that emerges from the interaction of affective, cognitive, and environmental factors.

Within classroom-based foreign language learning, WTC represents the final psychological step before overt communicative behavior (MacIntyre, 1994; MacIntyre et al., 2001). Accordingly, WTC has also been targeted in pedagogical intervention research. Munezane (2015) showed that training in visualization combined with goal-setting led to greater gains in learners’ confidence and WTC than visualization alone or no instructional intervention. Similarly, Yashima et al. (2018) found that reducing teacher control and encouraging student-initiated interaction increased group-level WTC while simultaneously lowering foreign language anxiety.

Technology-mediated and AI-assisted interventions have increasingly extended this line of inquiry to the domain of speaking. Zhang et al. (2024) demonstrated that technology-enhanced speaking practice significantly increased Chinese EFL learners’ WTC and reduced FLA over an intervention period, underscoring the affective responsiveness of WTC to instructional context. Tai and Chen (2023) found that interaction with a conversational AI agent was associated with higher WTC and reduced communication apprehension among EFL learners, suggesting that the low-stakes, self-paced nature of AI interaction may lower the affective threshold for initiating communication. Recent AI-mediated EFL research further suggests that learners’ AI-related self-efficacy, classroom anxiety, enjoyment, and trust can influence their willingness to communicate in AI-supported environments (Zhang et al., 2025; Zhang and Fan, 2026). However, reduced communication apprehension during AI interaction does not necessarily imply an equivalent reduction in broader classroom FLA. Therefore, the present study treats WTC improvement and FLA reduction as related but separable possibilities, rather than assuming that increased WTC must be accompanied by significantly lower FLA. However, existing AI-mediated WTC research has concentrated almost exclusively on EFL contexts and short-term designs, leaving open the question of whether AI-assisted practice can produce sustained gains in WTC among beginner learners of languages other than English, particularly in speaking-intensive LOTE courses where authentic communicative opportunities outside the classroom are scarce (Wen and Clément, 2003). The present study addresses this gap by examining WTC trajectories longitudinally in an AI-assisted Spanish speaking course among Chinese university learners.

### 2.3 FL emotions and WTC in foreign language learning

Research on emotions and WTC in foreign language learning has undergone a clear theoretical evolution. Early studies, grounded in traditional psychology and Krashen’s (1985) affective filter hypothesis, focused primarily on FLA and its inhibiting effect on learners’ WTC (MacIntyre et al., 2003; de Saint Léger and Storch, 2009). With the introduction of Positive Psychology (PP) into SLA (MacIntyre and Gregersen, 2012), subsequent research began to incorporate positive emotions, most notably FLE, and examined how enjoyment and anxiety jointly shape learners’ WTC (Khajavy et al., 2018; Dewaele and Pavelescu, 2019; Li et al., 2022; Feng et al., 2023; Lu, 2024).

More recently, research on emotions and WTC has been further informed by cross-disciplinary frameworks such as the broaden-and-build theory, control-value theory, and complex dynamic systems theory. From this perspective, emotions are conceptualized as dynamic, interacting, and context-sensitive processes rather than stable antecedents of communication. In addition to anxiety and enjoyment, discrete emotions including boredom (Li et al., 2023), pride (Khajavy and Lüftenegger, 2024), and shame (Teimouri, 2018) have attracted growing empirical attention, revealing a more differentiated emotional landscape underlying learners’ communicative readiness.

Despite this progress, empirical findings regarding the emotion-WTC relationship remain mixed, particularly with respect to boredom. Wang et al. (2021) reported that enjoyment positively predicted classroom WTC among Chinese EFL learners alongside the anticipated negative effects of anxiety, while also finding an unexpected positive association between boredom and WTC, a pattern partially replicated in subsequent work (Wang et al., 2024). In contrast, other studies identified boredom as a negative predictor of WTC (Kruk, 2022; Li et al., 2022), or found no significant relationship (Alrabai, 2022). Such inconsistencies suggest that the effects of emotions on WTC are likely contingent on contextual conditions and temporal dynamics rather than uniform across settings. They may also reflect measurement differences, as studies vary in whether boredom is assessed as a general classroom emotion, a task-specific state, or an activity-related experience, and whether WTC is measured as a broad communicative disposition or as classroom speaking readiness. In the present study, because FLB is conceptualized as monotony, disengagement, and loss of attention during Spanish speaking activities, it is expected to be negatively associated with WTC, although the strength of this relationship may vary across time and AI-mediated instructional conditions.

Recent research has increasingly emphasized the interrelations among emotions and their joint influence on WTC. For instance, Feng et al. (2023) reported that strong enjoyment effectively neutralized the negative impact of anxiety on WTC. However, other studies suggested that enhancing specific positive emotions does not necessarily lead to a reduction or elimination of negative emotions, as positive and negative emotions often coexist and exert simultaneous influences on learners’ communicative behavior (Dewaele and Pavelescu, 2019; Zhang et al., 2024; Lu, 2025). These findings underscore the dynamic and non-linear nature of emotional processes in L2 communication and highlight the need for research designs that capture how multiple emotions unfold and interact over time.

The emergence of AI-mediated speaking environments introduces a new instructional context in which the emotion-WTC relationship may operate differently. AI conversational agents offer low-pressure, self-paced interaction that removes the immediate evaluative presence of teachers and peers, potentially reconfiguring the affective conditions under which learners decide whether to communicate (Fryer et al., 2019; Kruk, 2022). Conceptually, this context connects the three theoretical perspectives guiding the present study. Positive Psychology provides the rationale for examining FLE, FLA, and FLB as distinct but coexisting emotional dimensions; Control-Value Theory suggests that AI-mediated speaking practice may shape these emotions by influencing learners’ perceived control, task value, and evaluative pressure; and the WTC framework explains why these emotional conditions are relevant to learners’ readiness to initiate communication. A small but growing body of research suggests that such environments can shift multiple affective variables simultaneously: Zhang et al. (2024) found that technology-enhanced speaking practice reduced FLA and increased WTC among Chinese EFL learners within a single semester, while Tai and Chen (2023) reported that AI-assisted interaction lowered communication apprehension and raised learners’ readiness to speak. These findings imply that the joint dynamics of emotions and WTC may be particularly responsive to AI-mediated instructional conditions. However, existing studies have not yet clarified how AI-assisted speaking practice may influence different emotional dimensions and how these emotional changes may relate to WTC over time.

Despite this emerging evidence, how the full affective triad of FLE, FLA, and FLB jointly changes with WTC under sustained AI-assisted speaking instruction remains unexamined. Existing studies have largely treated these constructs in isolation, examining emotions and WTC in separate lines of inquiry—and have relied on short-term or cross-sectional designs that cannot capture the delayed and interrelated changes among them over an instructional period (Lin and Wang, 2025; Zhang et al., 2025). A clearer conceptual account is therefore needed to examine whether AI-assisted speaking practice is associated with different patterns of change in FLE, FLA, and FLB, and whether these emotional changes are linked to learners’ WTC over time. This gap is particularly acute in LOTE speaking contexts, where the affective and communicative dynamics documented in EFL research may not straightforwardly generalize. The present study addresses this gap directly by tracking longitudinal changes in FLE, FLA, FLB, and WTC across an AI-assisted Spanish speaking course among Chinese university learners.

To address these gaps, and guided by this conceptual framing, the present study adopts a longitudinal quasi-experimental mixed-methods design to examine how AI-assisted speaking practice influences the longitudinal changes of FLE, FLA, and FLB, and how these shifts are longitudinally associated with WTC over time, among Chinese university learners of Spanish. Based on the literature reviewed above, FLE was expected to be positively associated with WTC and FLB was expected to be negatively associated with WTC, whereas FLA was expected to show weaker or non-significant associations with WTC in the AI-assisted speaking context. By integrating quantitative survey data with qualitative interview evidence, this study is guided by the following research questions:

*RQ1*. How does AI-assisted learning influence the longitudinal development of FL emotions (enjoyment, anxiety, and boredom) among Chinese university learners of Spanish?

*RQ2*. How does AI-assisted learning affect learners’ WTC in Spanish over time?

*RQ3*. What are the longitudinal relationships between FL emotions and learners’ WTC in an AI-assisted learning context?

*RQ4*. How do learners perceive and explain the emotional and communicative changes associated with AI use in Spanish learning, particularly with respect to possible delayed effects and the relative stability of anxiety?

## 3 Methods

### 3.1 The study design

A mixed-methods longitudinal quasi-experimental design was adopted in this study. A total of 69 beginner learners of Spanish from a Chinese university participated and were assigned to either an experimental group (EG) or a control group (CG) based on intact classes. The intervention was conducted over a 16-week period within a beginner-level elective Spanish course. Both groups followed the same course syllabus and instructional schedule. They were also taught by the same instructor and used the same teaching materials, classroom topics, and assessment schedule, thereby reducing potential instructor- and curriculum-related confounds. The only systematic difference between the two groups was the integration of TalkPal AI into the structured speaking-practice segments for the EG. The experimental group received regular classroom instruction supplemented with an AI-based conversational assistant, which was integrated into classroom-based speaking activities and used as an interlocutor for individual oral practice during designated instructional segments, whereas the control group followed the same instructional syllabus without AI support, relying instead on conventional classroom-based speaking activities.

Each instructional unit focused on a beginner-level communicative theme aligned with the course syllabus (e.g., self-introduction, daily routines, and personal preferences). Within this design, the AI-assisted speaking tasks were semi-structured and guided by standardized prompts to ensure consistency of task implementation across participants. During the intervention, EG students completed two structured TalkPal AI tasks during the designated speaking-practice segment of each weekly class. Each in-class AI-assisted segment lasted approximately five minutes. Across the semester, the required in-class AI component therefore consisted of 32 monitored sessions, corresponding to approximately 160 min of structured AI-assisted speaking practice. The AI intervention reported in this study referred only to these structured in-class AI-assisted speaking segments. Any voluntary out-of-class use of TalkPal AI was not required, monitored, or included as part of the intervention dosage. To support treatment fidelity, all EG students received the same weekly prompts and task sequence, and the instructor checked task completion during class through in-class observation. For the retained final sample, all EG students completed the required in-class AI-assisted tasks. However, the researchers did not have access to objective app-based usage logs; therefore, individual-level usage duration, interaction quality, or any voluntary out-of-class use could not be independently verified. During these activities, the AI system functioned as an interactive interlocutor by generating contingent follow-up questions and providing reformulations when needed, with feedback primarily delivered in an implicit manner to maintain a meaning-oriented communicative focus.

To capture changes over time, data were collected at three measurement points across the semester. At each wave, participants completed self-report questionnaires measuring WTC and key foreign language emotions, including enjoyment, anxiety, and boredom. This three-wave longitudinal design enabled the examination of both between-group differences and within-person developmental trajectories, as well as the dynamic relationships between AI-assisted interaction, emotional experiences, and WTC.

Ethical approval was obtained from the Ethics Committee of the first author’s institution. All participants provided written informed consent prior to participation in the study. Participation was voluntary, and participants were informed of their right to withdraw at any time without penalty. The study was conducted in accordance with the ethical standards of the institutional and national research committees and with the 1964 Declaration of Helsinki and its later amendments.

### 3.2 Research context and participants

The study was conducted at a university in eastern China. Participants were university students enrolled in a general elective course and were beginners in Spanish as a second foreign language, with no formal prior learning experience in Spanish, although they had previous foreign-language learning experience mainly through English instruction in prior schooling. They were taking an introductory Spanish-speaking course aimed at developing basic communicative competence. The course met once a week for a two-hour session over approximately 16 weeks during the semester. Instruction focused on fostering students’ foundational Spanish conversational abilities through communicative classroom activities.

All students enrolled in the course were invited to participate in the study, forming a convenience sample. Initially, 79 students took part in the study (EG = 40, CG = 39). After data screening and removal of incomplete responses, the final sample consisted of 69 students (EG = 35, CG = 34), resulting in an attrition rate of 12.7%. The 10 excluded participants were removed because they missed one or more measurement waves or provided incomplete questionnaire responses across the three waves. To examine whether attrition introduced systematic bias, baseline comparisons were conducted between completers and non-completers in terms of group distribution, gender, age, and T1 measures of FLE, FLA, FLB, and WTC. No statistically significant baseline differences were found. The mean age of the participants was 19.23 years (SD = 0.59), and the sample consisted of 52 female and 17 male students.

### 3.3 Data collection

To investigate the effects of AI-based interventions on beginning Spanish students’ FLE, FLA, FLB and WTC, a set of quantitative and qualitative instruments was employed. The study utilized an AI-based conversational assistant as the primary intervention platform. Data were subsequently collected through a battery of self-report questionnaires and a semi-structured interview protocol. The questionnaire package consisted of four validated scales, including three emotion-related scales measuring FLE, FLA, FLB, and one scale assessing learners’ WTC. Questionnaire data were collected at three time points during the semester: Week 4 (T1), Week 10 (T2), and Week 16 (T3), allowing for the examination of longitudinal changes in learners’ emotional states and communicative dispositions. At each measurement point, all participants completed self-report measures of FLE, FLA, FLB, and WTC.

To enhance measurement comparability across groups, the same construct domains, item stems, response format, number of response options, and scoring procedures were retained for the EG and the CG across all three waves. All core questionnaire items referred to in-class Spanish speaking activities. For the EG, these activities included AI-mediated speaking tasks; for the CG, they referred to conventional classroom speaking activities following the same syllabus. Thus, the questionnaires were designed to assess the same emotional and communicative constructs under the respective instructional conditions. Nevertheless, because the concrete activity referents differed across conditions, potential construct nonequivalence cannot be completely ruled out and is acknowledged as a limitation. Following the final wave of data collection, a subset of participants from the EG (*n* = 9) participated in semi-structured interviews to explore their experiences with AI-assisted classroom interaction. The qualitative component focused exclusively on the EG to gain an in-depth understanding of the experiential mechanisms underlying their longitudinal affective and communicative changes. The interview protocol focused on these learners’ experiences of AI-mediated communication and their perceptions related to WTC during interactions with the AI conversational assistant.

#### 3.3.1 Intervention tool: TalkPal AI

The intervention device employed in this study was an AI-based conversational application, TalkPal AI, designed to support foreign language learners in developing speaking proficiency and communicative competence. The application integrates speech recognition and natural language processing technologies to simulate interactive conversations and provide learners with immediate feedback during oral practice. Through a range of conversational scenarios, learners are encouraged to engage in target-language communication in everyday, academic, and situational contexts, while receiving instant feedback on pronunciation accuracy, fluency, and language appropriateness.

In addition to corrective feedback, TalkPal AI offers model responses and alternative expressions that allow learners to refine their utterances and expand their lexical and structural repertoire. The application supports individualized and self-paced learning by enabling repeated speaking practice without time constraints or fear of negative evaluation, which is particularly beneficial for beginning-level learners. Learners may select or generate topics aligned with their interests and proficiency level, thereby promoting sustained engagement and learner autonomy during speaking practice.

Furthermore, TalkPal AI provides a psychologically safe environment for oral production, as learners can practice speaking as frequently as needed without the social pressure typically associated with classroom interaction. This affordance aligns with communicative language teaching principles that emphasize meaningful communication, learner-centered interaction, and confidence-building through authentic language use. By lowering affective barriers and increasing opportunities for individualized speaking practice, TalkPal AI serves as an appropriate intervention tool for examining the emotional and communicative effects of AI-assisted language learning. Although TalkPal AI allows flexible and individualized practice, the present intervention used the application as a structured classroom-based interlocutor rather than as an open-ended out-of-class learning requirement. The specific intervention dosage was therefore defined by the monitored in-class speaking-practice segments described above.

#### 3.3.2 The FLE scale

Foreign Language Enjoyment (FLE) was measured using a 12-item scale adapted from Jin and Qin (2024) for Chinese students. Items were adapted to the Spanish LOTE context by focusing on Spanish speaking classes rather than general learning. Specifically, references to English learning or speaking English in the original items were replaced with Spanish learning or speaking Spanish, and classroom references were contextualized as in-class Spanish speaking activities. The adapted items were reviewed by bilingual researchers to ensure that the Spanish-context wording preserved the meaning of the original constructs. The same item stems and response format were used for both groups, with the activity referent aligned with each group’s instructional condition. To enhance measurement comparability, both groups completed the same items. However, the EG was instructed to evaluate AI-mediated practice, while the CG referred to regular classroom activities. The scale assesses learners’ positive emotional experiences in foreign language learning, with responses rated on a five-point Likert scale. An example item is: “I feel confident when speaking Spanish during the in-class activities.” Excellent internal consistency across the three waves was demonstrated, with Cronbach’s alpha coefficients of 0.907 (T1), 0.953 (T2), and 0.945 (T3).

#### 3.3.3 The short-form FLCA scale

Foreign Language Anxiety (FLA) was measured using eight items extracted from the original Foreign Language Classroom Anxiety Scale developed by Horwitz et al. (1986), following the short-form FLCA measure used by Dewaele and MacIntyre (2014). The scale captures learners’ anxiety experienced in foreign language classroom contexts, particularly feelings of nervousness and tension during language use. Consistent with the adaptation of the FLE scale, items were modified to focus on Spanish speaking classes to enhance context-sensitivity and measurement comparability The scale includes items reflecting common sources of foreign language anxiety, such as fear of speaking in front of others and experiencing anxiety even when well prepared. A sample item is: “I feel anxious even though I am fully prepared for Spanish class.” All items were rated on a five-point Likert scale. The scale was translated and back-translated by two bilingual experts to ensure linguistic accuracy and demonstrated acceptable internal consistency across the three waves, with Cronbach’s alpha coefficients of 0.770 (T1), 0.780 (T2), and 0.797 (T3).

#### 3.3.4 The short-form FLB scale

Foreign Language Boredom (FLB) was measured using the eight-item short form of the Foreign Language Classroom Boredom Scale (S-FLCBS), adapted from the English classroom boredom scale derived from a subscale of the broader Foreign Language Boredom Scale developed by Li et al. (2023) for Chinese students. The scale is a unidimensional instrument designed to assess students’ experience of boredom in foreign language classroom settings. Following the same adaptation logic as the FLE and FLA scales, items were contextualized to focus on Spanish speaking during class rather than the general classroom environment. The scale captures learners’ feelings of monotony, disengagement, and loss of attention during language learning activities. All items were rated on a five-point Likert scale. A sample item is: “My mind starts to wander during the assigned Spanish speaking activities in class.” The scale demonstrated excellent internal consistency across the three waves, with Cronbach’s alpha coefficients of 0.935 (T1), 0.957 (T2), and 0.974 (T3).

#### 3.3.5 The WTC scale

Willingness to Communicate (WTC) in Spanish was measured using a 10-item scale adapted from Peng and Woodrow (2010), whose measure was based on Weaver’s (2005) classroom WTC scale. The scale was originally developed to assess students’ willingness to speak English in classroom contexts. Following the same adaptation logic as previous scales, items were contextualized to focus on Spanish speaking during class to enhance context-sensitivity and measurement comparability. The scale evaluates learners’ readiness to initiate or participate in oral communication during Spanish classroom activities, with responses rated on a five-point Likert scale. A sample item is: “I am willing to speak Spanish during the assigned speaking activities.” The questionnaire was translated and back-translated by two bilingual experts to ensure linguistic accuracy. The scale demonstrated excellent internal consistency across the three waves, with Cronbach’s alpha coefficients of 0.914 (T1), 0.947 (T2), and 0.954 (T3).

#### 3.3.6 A semi-structured interview

Semi-structured interviews were conducted to provide explanatory qualitative evidence for the quantitative findings. Because the qualitative component was intended to explain learners’ experiences with the AI-assisted intervention, interviews were conducted with participants from the EG. Using purposive sampling, nine EG participants were invited to participate in semi-structured interviews (see Appendix A) at the end of the intervention. Participants were selected to reflect variation in Spanish proficiency, classroom participation, and perceived engagement with AI-assisted speaking practice. The interviews, each lasting 20–30 min, were conducted in Mandarin Chinese to ensure communicative clarity and were audio-recorded with participants’ prior informed consent. Subsequently, the recordings were transcribed verbatim and translated into English for qualitative analysis. The interview questions broadly focused on learners’ affective experiences, perceived influences on WTC, challenges encountered during human–AI interaction, and perceived differences between communicating with AI chatbots and with human interlocutors. During preliminary coding, no substantially new themes emerged from the final interviews, suggesting thematic saturation for the explanatory purposes of this qualitative component.

### 3.4 Data analysis

All quantitative analyses were conducted using R (version 4.4.0). Descriptive statistics were first calculated to summarize participants’ levels of FLE, FLB, FLA, and WTC across the three measurement points (T1, T2, and T3). The normality of the data was examined using Q-Q plots. To assess the potential influence of common method bias in the self-report quantitative data, Harman’s single-factor test was conducted separately for the item-level measures at each wave.

Independent-samples t-tests were conducted to examine baseline differences between the experimental group (EG) and the control group (CG) at T1. To account for the repeated-measures structure of the three-wave data, linear mixed-effects models (LMMs) were fitted for each outcome, with Time, Group, and Time × Group as fixed effects and participant ID as a random intercept. *A priori* planned contrasts based on LMM-estimated marginal means were then conducted to examine change differences across T1–T2 and T1–T3, as well as endpoint group differences at T2 and T3. Finally, Pearson correlation analyses of change scores were conducted to explore longitudinal associations between changes in foreign language emotions and WTC.

The qualitative interview data were analyzed using a theory-informed thematic coding approach. Guided by the research questions, the first author read the transcripts repeatedly and noted segments related to learners’ emotional experiences, communicative willingness, and perceptions of AI-mediated speaking practice. Coding was not restricted to predefined constructs; additional codes and themes were generated inductively from participants’ accounts. Recurrent and conceptually related codes were then refined into broader interpretive themes. To enhance trustworthiness, the coding framework, theme labels, and representative excerpts were reviewed through peer debriefing with another researcher, and revisions were made where necessary to improve the clarity and coherence of the thematic interpretation.

## 4 Results

### 4.1 Between-group comparisons of Spanish-learning FLE, FLB, FLA, and WTC

**Figure 1:**
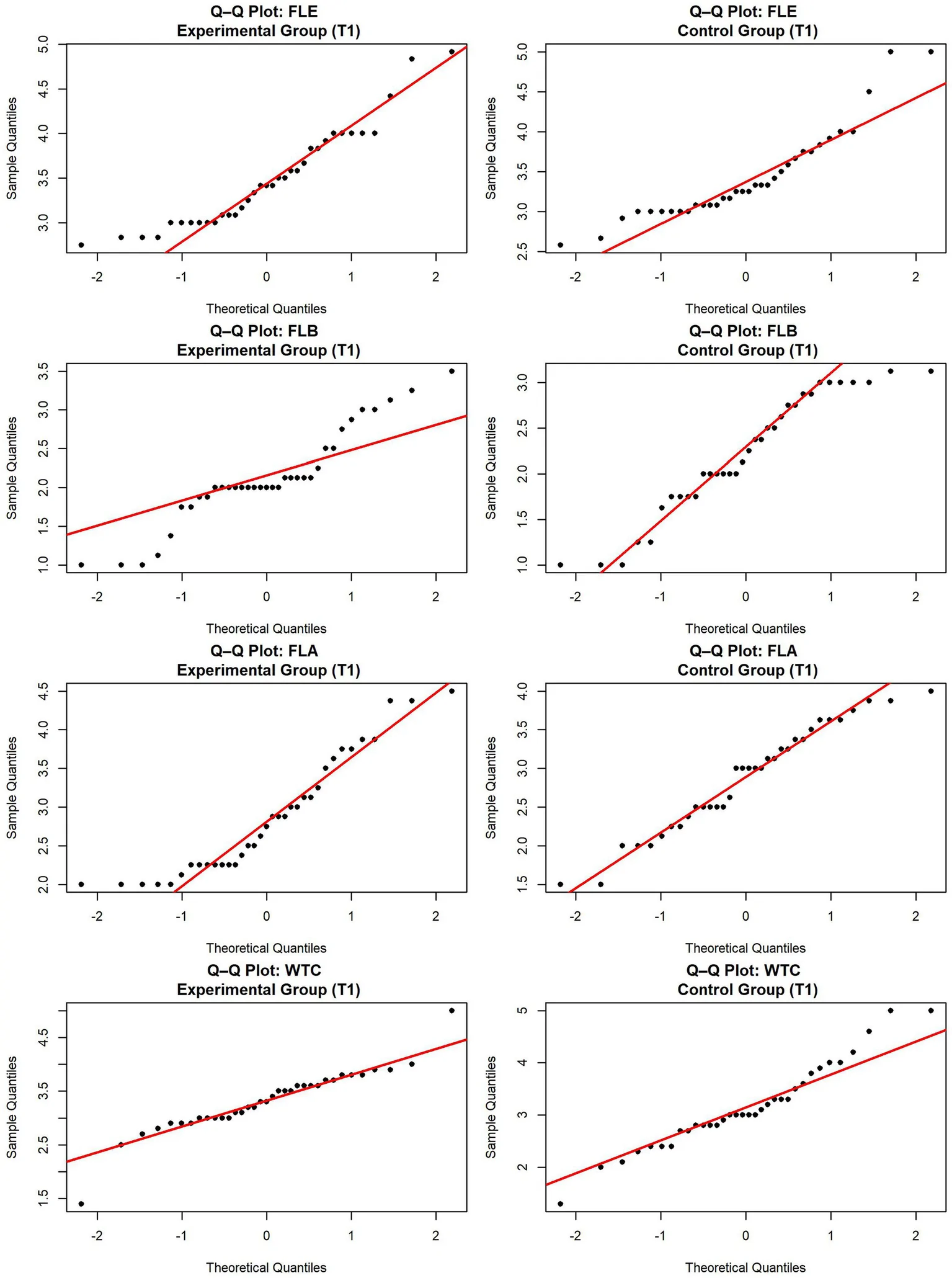
Normal Q-Q plots of FLE, FLB, FLA, and WTC for EG and CG at T1.

Mean scores were calculated to present participants’ levels of FLE, FLB, FLA and WTC across three measurement time points (pre-intervention, mid-intervention, and post-intervention) throughout the AI-assisted speaking intervention. Table 1 presents the descriptive statistics for the EG and the CG across T1, T2, and T3, together with baseline group comparisons at T1. Both groups exhibited moderate levels of FLE (EG M = 3.49, CG M = 3.43) and WTC (EG M = 3.32, CG M = 3.15), whereas their mean scores for FLB (EG M = 2.12, CG M = 2.21) and FLA (EG M = 2.87, CG M = 2.87) were below the scale midpoint. Q-Q plots in Figure 1 depicted approximately normal distribution for FLE, FLB, FLA, and WTC in both groups.

**Table 1:** Descriptive statistics and baseline group comparisons for FLE, FLB, FLA, and WTC.

Variable	Group	T1	T2	T3	Baseline t	Baseline p
FLE	EG	3.49 (0.55)	3.56 (0.71)	3.79 (0.63)	−0.45	0.651
	CG	3.43 (0.57)	3.40 (0.65)	3.52 (0.61)	—	—
FLB	EG	2.12 (0.62)	2.11 (0.59)	1.91 (0.66)	0.65	0.521
	CG	2.21 (0.66)	2.29 (0.79)	2.29 (0.76)	—	—
FLA	EG	2.87 (0.76)	2.82 (0.73)	2.72 (0.79)	0.00	0.999
	CG	2.87 (0.69)	2.91 (0.61)	2.90 (0.68)	—	—
WTC	EG	3.32 (0.58)	3.47 (0.72)	3.68 (0.60)	−0.97	0.335
	CG	3.15 (0.82)	3.14 (0.77)	3.32 (0.82)	—	—

N (EG) = 35, N (CG) = 34. Values for T1, T2, and T3 are means with standard deviations in parentheses.

Subsequently, independent-samples t-tests were conducted to examine whether there were any pre-intervention differences between the EG and the CG in FLE, FLB, FLA, and WTC. No statistically significant differences were observed between the EG and the CG in any of the four variables at Time 1 (all *p* > 0.05), indicating that the two groups were comparable prior to the intervention (see Table 1).

**Figure 2:**
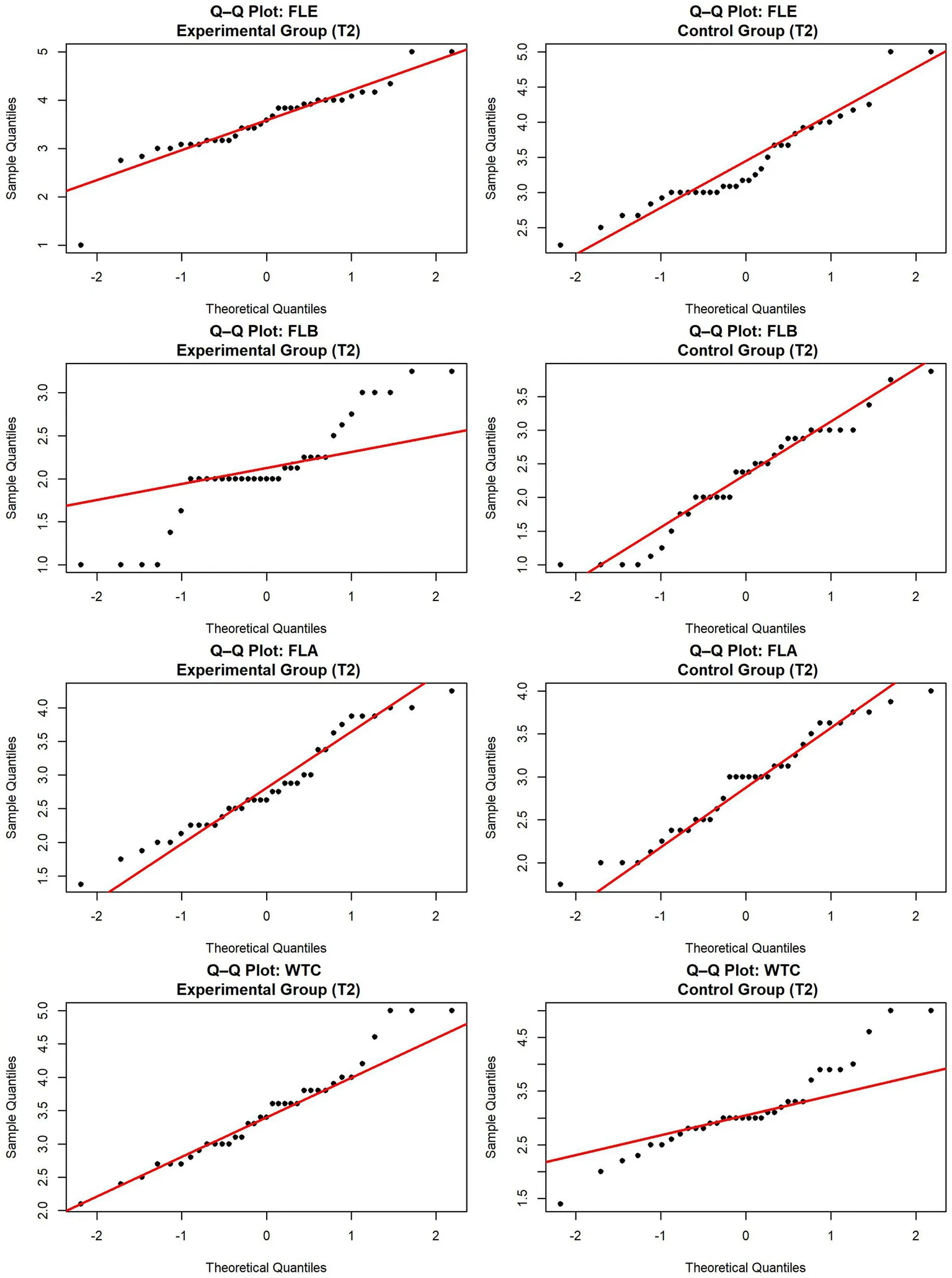
Normal Q-Q plots of FLE, FLB, FLA, and WTC for EG and CG at T2.

**Figure 3:**
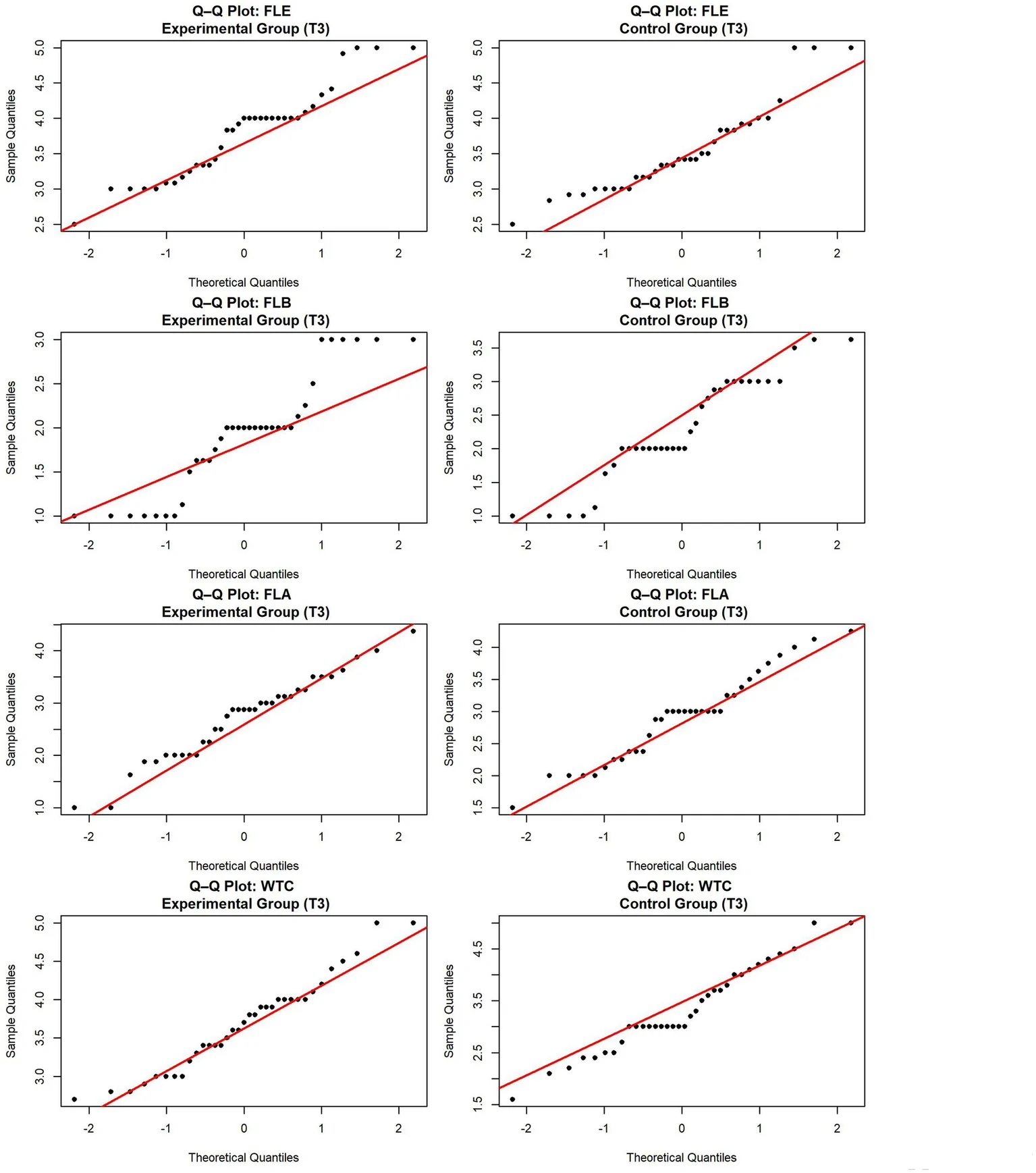
Normal Q-Q plots of FLE, FLB, FLA, and WTC for EG and CG at T3.

A comparison of mean scores at Time 2 (during the intervention) revealed that the EG demonstrated higher levels of FLE and WTC than the CG, whereas the EG reported lower levels of FLB and FLA compared with the CG. A consistent pattern emerged at Time 3 (post-intervention). Specifically, the EG showed higher mean scores in FLE and WTC and lower mean scores in FLB and FLA than the CG. Q-Q plots in Figures 2, 3 for both the EG and the CG at each time point suggested approximately normal distributions.

### 4.2 Longitudinal changes in FLE, FLB, FLA, and WTC at T1, T2, and T3

**Figure 4:**
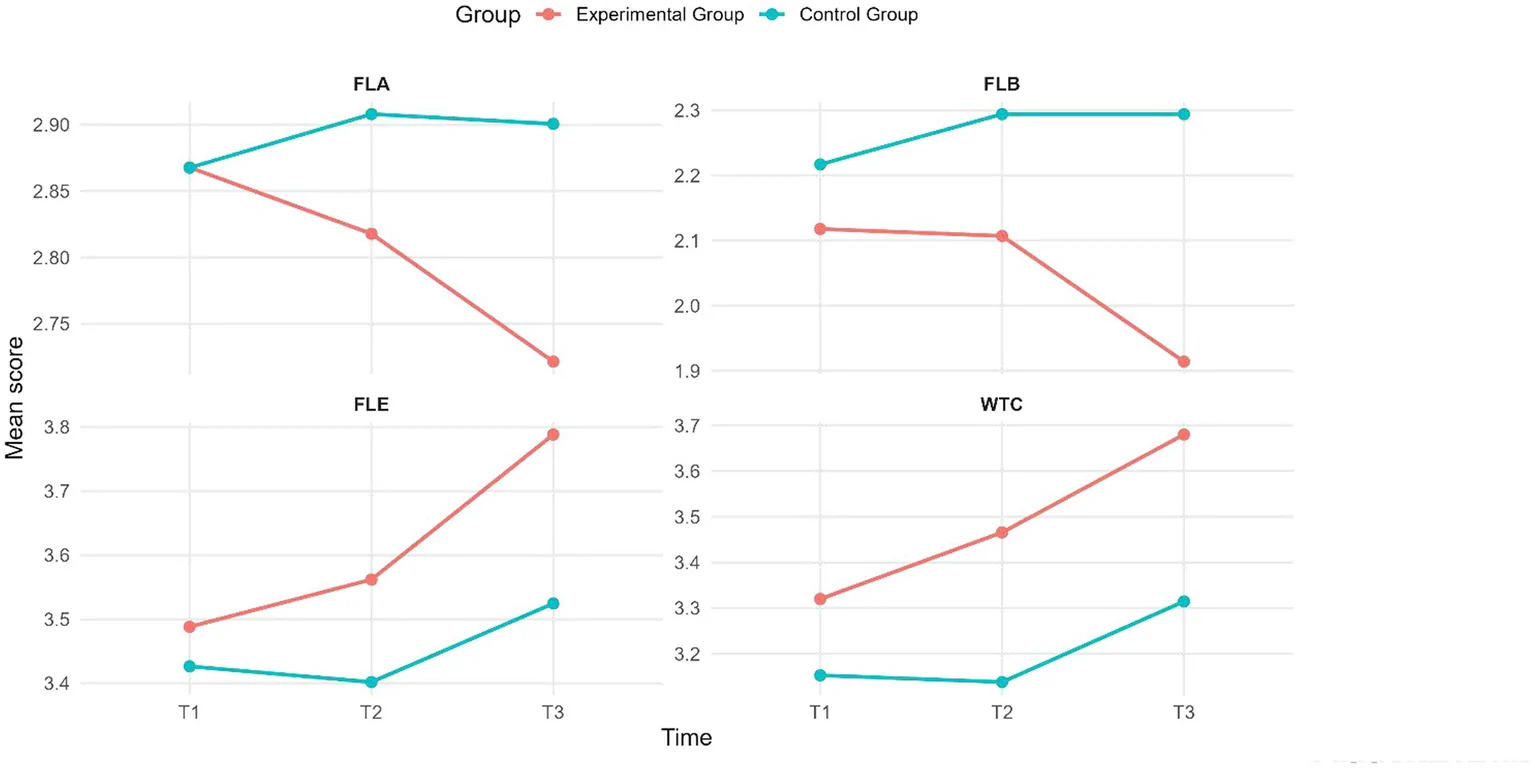
The changes of FLE, FLB, FLA, and WTC over time in EG and CG.

Figure 4 illustrates the trajectories of FLE, FLB, FLA, and WTC across three time points (T1-T3) for the EG and the CG. As shown in the figure, both groups exhibited an overall increase in FLE and WTC over time; however, the growth was more pronounced for the EG, particularly from T2 to T3. In contrast, FLA and FLB demonstrated divergent patterns between groups. The EG showed a steady decrease in both anxiety and boredom across the intervention period, whereas the CG remained relatively stable with only minor fluctuations. Notably, group differences became more apparent at T3, with the EG reporting higher levels of positive affective variables (FLE and WTC) and lower levels of negative affective variables (FLA and FLB) compared to the CG.

To examine longitudinal changes across the three measurement points while accounting for the repeated-measures structure of the data, linear mixed-effects models (LMMs) were fitted for FLE, FLB, FLA, and WTC. For each outcome, Time, Group, and the Time × Group interaction were included as fixed effects, and participant ID was included as a random intercept. Degrees of freedom were estimated using the Satterthwaite method.

As shown in Table 2, significant main effects of Time were observed for FLE and WTC, indicating overall changes in these two variables across the semester. The Time × Group interaction approached significance for FLB, suggesting a possible group-specific pattern in boredom reduction. No other Time × Group interactions reached statistical significance.

**Table 2:** Three-wave linear mixed-effects model results.

Variable	Effect	df	F	p	ηp^2^
FLE	Time	2,138	6.66	0.002	0.088
	Group	1,69	1.52	0.221	0.022
	Time × Group	2,138	1.44	0.240	0.020
FLB	Time	2,138	1.13	0.326	0.016
	Group	1,69	2.40	0.126	0.034
	Time × Group	2,138	2.43	0.092	0.034
FLA	Time	2,138	0.72	0.490	0.010
	Group	1,69	0.32	0.572	0.005
	Time × Group	2,138	1.46	0.236	0.021
WTC	Time	2,138	11.62	< 0.001	0.144
	Group	1,69	3.23	0.077	0.045
	Time × Group	2,138	1.75	0.178	0.025

*N* (EG) = 35, *N* (CG) = 34.

### 4.3 Planned longitudinal changes in FLE, FLB, FLA, and WTC at T1, T2 and T3

*A priori* planned contrasts were conducted based on the LMM-estimated marginal means to examine whether group differences in change emerged over specific phases of the intervention. The planned contrasts also provided estimates of phase-specific change differences and endpoint group differences in the original scale metric. Planned interaction contrasts tested whether the EG changed more than the CG during the early phase of the intervention (T1–T2) and across the full intervention period (T1–T3). Planned endpoint group contrasts further examined whether the EG and the CG differed at T2 and T3.

As shown in Table 3, none of the T1–T2 change differences reached statistical significance, indicating that the EG and the CG did not differ significantly in short-term change during the early phase of the intervention. Over the full T1–T3 period, the change difference for FLB was significant, indicating a greater reduction in boredom in the EG than in the CG. The remaining T1–T3 change differences did not reach conventional significance, although they were generally in the expected direction.

**Table 3:** Planned interaction contrasts for change differences between the EG and CG.

Variable	Change difference	Estimate (ΔEG − ΔCG)	SE	t	p
FLE	T1–T2	0.098	0.121	0.82	0.416
	T1–T3	0.202	0.121	1.67	0.096
FLB	T1–T2	−0.088	0.132	−0.67	0.507
	T1–T3	−0.281	0.132	−2.12	0.035
FLA	T1–T2	−0.090	0.107	−0.85	0.398
	T1–T3	−0.180	0.107	−1.68	0.094
WTC	T1–T2	0.160	0.114	1.40	0.163
	T1–T3	0.198	0.114	1.74	0.085

As shown in Table 4, no significant endpoint group differences were observed at T2. At T3, the EG reported significantly lower FLB and significantly higher WTC than the CG. The T3 group difference in FLE approached significance, whereas the T3 group difference in FLA remained non-significant. These results suggest that delayed group differences were most clearly reflected in FLB, with additional endpoint evidence for WTC at T3.

**Table 4:** Planned endpoint group contrasts at T2 and T3.

Variable	Time	Estimate (EG − CG)	SE	t	p
FLE	T2	0.160	0.150	1.07	0.289
FLE	T3	0.264	0.150	1.76	0.082
FLB	T2	−0.187	0.164	−1.14	0.257
FLB	T3	−0.380	0.164	−2.31	0.023
FLA	T2	−0.090	0.172	−0.53	0.601
FLA	T3	−0.179	0.172	−1.04	0.300
WTC	T2	0.327	0.175	1.87	0.064
WTC	T3	0.365	0.175	2.09	0.039

### 4.4 Longitudinal associations between changes in FL emotions and WTC

Change-score correlation analyses were conducted to examine the longitudinal associations between changes in foreign language emotions and changes in willingness to communicate (WTC) across the intervention period.

As shown in Table 5, during the early phase of the intervention (T1–T2), changes in foreign language emotions and WTC exhibited limited and inconsistent associations in both the experimental and control groups, with only isolated correlations emerging among emotional variables.

**Table 5:** Correlations among T1–T2 changes in FL emotions and WTC.

Group	Variables	ΔFLE	ΔFLB	ΔFLA	ΔWTC
EG	ΔFLE	—			
	ΔFLB	−0.10 (0.58)	—	—	
	ΔFLA	0.02 (0.91)	0.41 (0.015)*	—	
	ΔWTC	−0.10 (0.57)	−0.19 (0.26)	−0.29 (0.09)	—
CG	ΔFLE	—			
	ΔFLB	−0.50 (0.017)*	—		
	ΔFLA	−0.28 (0.11)	0.07 (0.70)	—	
	ΔWTC	0.34 (0.24)	−0.15 (0.40)	0.02 (0.92)	—

In contrast, Table 6 indicates that over the full intervention period (T1–T3), a clearer relational pattern emerged in the experimental group, where changes in foreign language enjoyment were positively associated with changes in WTC, whereas no corresponding emotion–WTC associations were observed in the control group.

**Table 6:** Correlations among T1–T3 changes in FL emotions and WTC.

Group	Variables	ΔFLE	ΔFLB	ΔFLA	ΔWTC
EG	ΔFLE	—			
	ΔFLB	−0.08 (0.65)	—		
	ΔFLA	−0.10 (0.58)	0.29 (0.09)	—	
	ΔWTC	0.43 (0.011)*	−0.19 (0.27)	−0.22 (0.21)	—
CG	ΔFLE	—			
	ΔFLB	−0.50 (0.015)*	—		
	ΔFLA	−0.12 (0.49)	0.08 (0.67)	—	
	ΔWTC	0.26 (0.13)	−0.20 (0.26)	−0.13 (0.45)	—

### 4.5 Qualitative results: explanatory insights from interviews

The qualitative findings were used to further explain the quantitative patterns, particularly the absence of short-term change differences during T1–T2 and the delayed pattern observed across the full intervention period, most clearly reflected in FLB and further supported by the T3 endpoint difference in WTC. The thematic analysis identified five recurrent themes that were inductively refined from participants’ accounts and interpreted in relation to the study’s focus on foreign language emotions and WTC: psychologically safe rehearsal space, early calibration difficulties, growing communicative control and agency, persistent evaluation-related anxiety, and perceived limits of AI-mediated interaction. These themes organize the qualitative findings below and help contextualize the longitudinal quantitative results. Appendix B summarizes the thematic coding framework, including themes, subthemes, and illustrative codes. Many participants emphasized that interacting with TalkPal AI during designated classroom speaking segments created a low-evaluative and supportive interactional space. Unlike whole-class or peer-based oral activities, AI-mediated interaction reduced learners’ concerns about negative evaluation while still requiring active language production. As one participant explained, “*When practicing with AI in class, I wasn’t as nervous as when speaking in front of my classmates, because I did not worry about them thinking I spoke badly, nor did I worry about seeing their puzzled looks.*” (EG-4).

Another participant highlighted the role of implicit feedback, stating that “*The AI corrected me naturally during the conversation, so I did not feel interrupted or embarrassed.*” (EG-8).

However, this supportive atmosphere did not immediately translate into heightened enjoyment. At the beginning of the semester, learners experienced what can be described as communication breakdowns (Varonis and Gass, 1985). Learners were primarily preoccupied with processing the unpredictable AI input and struggling to mobilize their limited linguistic resources to produce even basic utterances. One participant (EG-7) reflected that *“At the beginning, I found it difficult to enjoy talking to AI because I have limited language skills and my attention was mainly focused on completing tasks.”* This procedural friction represented a temporary deficit in both control and value. This helps explain why no significant T1–T2 change differences emerged in the planned contrasts.

This transition suggests that the reduction in communication breakdowns (Varonis and Gass, 1985) liberated attentional resources, enabling learners to shift from rigid task-following to spontaneous, off-script communication. This newfound communicative agency allowed learners to move beyond external task requirements and begin appreciating the task’s intrinsic value, the inherent pleasure of authentic self-expression. Consequently, this shift from mechanical task completion to exploratory communication may help account for the later upward tendency in FLE and the decline in FLB, as the perception of monotony was replaced by creative discovery.

Several participants further described a gradual increase in WTC during AI-mediated classroom practice. Repeated interaction with the AI helped learners become more accustomed to expressing themselves in Spanish, reducing hesitation and fostering communicative confidence. The following extracts illustrate this progression:

“*Over time I was able to communicate with AI more smoothly, which made me realize that I had become stronger in the process. I also tried to break free from the teacher’s task framework, started to say what I really wanted to say, and explored topics on my own.*” (EG-1).

“*After practicing with the AI several times in class, I felt more willing to speak because I was more used to expressing myself.*” (EG-2).

These accounts suggest that growing familiarity and perceived communicative control underpinned the later improvement in WTC, consistent with the endpoint contrast showing higher WTC in the EG than in the CG at T3.

In contrast, FLA remained statistically stable across time points. Interview data further indicated that anxiety was primarily associated with formal evaluation and instructor-led assessment. As one learner noted, “*Practicing with AI is actually fun and less stressful, but when I think about the oral exam, I still get worried.*” (EG-6).

This pattern suggests that while AI-supported practice reduced nervousness during rehearsal, concerns related to high-stakes oral assessment persisted throughout the semester. This distinction between practice-related comfort and evaluation-related anxiety provides a qualitative explanation for the statistical stability of FLA across time points. Learners appeared to perceive AI as a supportive rehearsal partner, but not as a substitute for high-stakes oral performance contexts.

A final recurrent theme concerned learners’ perceived differences between AI-mediated and human-mediated interaction. Although learners generally viewed TalkPal AI as a useful rehearsal partner, some noted that the novelty of AI interaction declined over time and that its responses could become predictable. “*By the end of the semester, the novelty had worn off. After using it for a while, I even felt like I could guess what its replies would be. It was a real robot, knowledgeable but lacking in emotion, and it did not feel like I spoke to a real person.*” (EG-3).

“*The AI is helpful for practice, but it cannot fully replace the emotional exchange we get from communicating with our teacher or classmates. I prefer real people because the interaction feels more meaningful and vibrant.*” (EG-9).

These accounts suggest that emotional authenticity may shape the long-term sustainability of AI-mediated speaking practice. Rather than replacing human interaction, AI may be most useful as a scaffolded rehearsal space that prepares learners for subsequent communication with teachers and peers.

## 5 Discussion

This study examined the impact of AI-mediated speaking practice on Chinese university learners of Spanish, focusing on the longitudinal trajectories of FLE, FLA, FLB, as well as their relationships with WTC. A combination of quantitative survey data and qualitative interview evidence allowed for a more fine-grained, process-oriented account of how AI interaction may shape learners’ affective and communicative experiences over time. Compared with prior cross-sectional studies conducted primarily in English-learning contexts, this longitudinal investigation in a Spanish LOTE setting suggests that the affective and communicative effects of AI-mediated speaking practice may emerge gradually rather than immediately. However, this delayed pattern was not uniform across all outcomes: it was most clearly reflected in the reduction of FLB, further supported by the endpoint difference in WTC at T3, and only suggested as a trend for FLE, while FLA remained statistically stable. The planned contrast estimates further supported this interpretation, showing that the clearest phase-specific change difference emerged for FLB over T1–T3, whereas WTC was more evident as an endpoint group difference at T3. Regarding the first research question, the findings indicate that AI-mediated speaking practice was most clearly associated with a delayed reduction in FLB, while also showing a more tentative, directionally consistent pattern of increased FLE over time. FLA remained relatively stable. Given the modest sample size, this null pattern should be interpreted cautiously as an absence of statistically detectable FLA change in the present study, rather than as conclusive evidence that AI-mediated speaking practice has no anxiety-related effect. Early in the intervention, unfamiliarity with AI feedback mechanisms created interactional uncertainty, which constrained learners’ perceived control. However, as the environment became more predictable and linguistic resources accumulated, learners’ communicative self-efficacy appeared to increase. This transition toward proactive engagement not only enhanced their task-related agency but also allowed them to recognize the intrinsic communicative value of the AI-mediated task, namely, the immediate gratification of functional communication. Collectively, these shifts in control and value appraisals may help explain the observed reduction in FLB and the more tentative upward tendency in FLE over time. These findings are broadly consistent with the Control-Value Theory (Pekrun, 2006), highlighting that by making the achievement environment more nonjudgmental and autonomy-supportive, AI-mediated interaction may support perceived control and task-intrinsic value, thereby reducing deactivating emotions such as FLB and potentially supporting positive activating emotions such as FLE. The stability of FLA may reflect the persistence of evaluation-related apprehension in high-stakes assessment contexts, a pattern also reported in previous studies in Asian educational contexts (Wen and Clément, 2003; Jiang and Dewaele, 2019). Unlike Tai and Chen’s (2023) finding that AI interaction reduced communication apprehension, the present results suggest that AI-mediated practice may ease rehearsal-related pressure without substantially reducing anxiety tied to formal assessment and limited linguistic resources.

In terms of the second research question, the study provides partial evidence for a delayed improvement in WTC. Although the endpoint group difference in WTC at T2 was in the expected direction but did not reach conventional significance, the experimental group reported significantly higher WTC than the control group at T3. As learners became more accustomed to AI interaction, they reported greater communicative confidence and willingness to extend conversations beyond prescribed prompts. This pattern suggests that repeated, low-pressure interaction may have supported the development of perceived communicative control and later communicative readiness. This finding is also consistent with socio-cognitive perspectives on WTC, which emphasize the role of situational confidence and interactional context in shaping communicative readiness (Tai and Chen, 2023).

Regarding the third research question, the longitudinal relationships between FL emotions and WTC suggest that changes in FLE were positively associated with changes in WTC in the experimental group over the full intervention period, whereas changes in FLB and FLA showed no consistent associations with changes in WTC. The cumulative nature of emotional change appears noteworthy: improvements in enjoyment gradually were associated with learners’ communicative engagement over time, whereas anxiety associated with high-stakes evaluation remained largely unaffected by AI practice. This underscores the temporal dynamics of emotion and WTC associations and aligns with previous findings on the importance of affective factors in promoting L2 communication (Dewaele and Dewaele, 2018; Dewaele and Pavelescu, 2019). These results suggest that gains in willingness to communicate in AI-supported environments may be more closely tied to the strengthening of positive affect than to changes in anxiety, which remained relatively stable across the intervention. Finally, concerning the fourth research question, qualitative data suggest that learners perceived AI as a supportive rehearsal partner that complemented traditional instruction. Key affordances included implicit feedback, task autonomy, and a low-pressure environment, which collectively facilitated procedural habituation and subsequent affective and communicative adjustment. Learners acknowledged limitations, such as the predictability of AI responses and reduced emotional authenticity, yet emphasized that these did not outweigh the benefits for engagement and communicative practice. These insights provide a possible explanatory account for the observed quantitative patterns, highlighting how AI-mediated practice may gradually reduce boredom, support later communicative willingness, and provide a possible basis for increased enjoyment even without fully replicating high-stakes or human-mediated interactions.

These findings suggest that AI-mediated conversational practice may facilitate a delayed but gradual shift in beginner LOTE learners’ emotional landscapes. Rather than serving as an immediate anxiety-reduction tool, the integration of AI appeared to support a gradual affective pathway in which benefits became more visible after learners developed greater interactional predictability and communicative control. From a theoretical perspective, this study provides tentative support for the applicability of Control-Value Theory to the domain of AI-supported LOTE speaking. In Pekrun’s (2006) original formulation, achievement emotions are shaped by learners’ appraisals of control and value in achievement activities. The present study does not revise this core mechanism; rather, it extends its application by highlighting how control and value appraisals may accumulate through repeated AI-mediated interaction. The results suggest that AI-mediated interaction may function as a longitudinal contextual affordance that gradually supports changes in learners’ control-value appraisals; specifically, by providing a predictable and responsive environment, the technology may support perceived control and may also increase the intrinsic value of the speaking task by ensuring successful communicative outcomes. This process may be more clearly reflected in the mitigation of deactivating emotions such as FLB, while also pointing to a possible role in supporting positive activating emotions such as FLE.

The present study offers tentative evidence for a temporal reading of Control-Value Theory, suggesting that learners’ emotional development in AI-mediated language learning is not immediate but progressively constructed through repeated interaction over time. This delayed pattern does not necessarily challenge CVT’s emphasis on the proximal role of appraisals; rather, it suggests that momentary appraisals may need to accumulate across repeated successful interactions before they become visible as measurable changes in semester-level emotional trajectories. Unlike the rapid emotional relief reported in short-term EFL studies (e.g., Zhang et al., 2024), our longitudinal evidence points to a delayed affective pattern, reflecting the unique interactional challenges and “motivational drift” often observed in LOTE contexts (Dewaele and MacIntyre, 2022; Wen and Clément, 2003). In the present study, this motivational drift was reflected in learners’ early task-completion orientation, limited linguistic resources, and some later comments that AI responses became less novel and more predictable. This suggests that AI may function as a temporal affordance that requires a “calibration period” to overcome initial communication breakdowns (Varonis and Gass, 1985) before emotional benefits become visible. From a skill-acquisition perspective (DeKeyser, 2007), this period may involve the gradual routinization of interactional procedures, allowing attention to shift from task execution to meaning-focused self-expression. The 16-week intervention appeared sufficient to capture partial habituation and emerging communicative control, but not necessarily full automaticity or stable long-term affective change. Specifically, as learners moved from a reactive state to one of situational command, the dual optimization of perceived control and task-intrinsic value may have supported the observed reduction in FLB and, to a lesser extent, the upward tendency in FLE. This is compatible with, but does not directly establish, the state-to-trait transformation of enjoyment hypothesized by Li and Dewaele et al. (2024), where repeated positive experiences during in-class tasks accumulate into a stable disposition. However, the three-wave design of the present study cannot directly separate state and trait components of enjoyment. Future research using more intensive repeated measures and latent state–trait models would be needed to test this transformation more directly. Furthermore, the sustained reduction in FLB aligns with the broaden-and-build theory (MacIntyre and Gregersen, 2012), suggesting that as AI became a meaningful interlocutor, the expanded psychological resources may have supported a more engaging and less monotonous learning experience across the intervention.

The study offers several practical implications for foreign language pedagogy. First, to mitigate FLB and potentially support FLE, instructors may design AI-assisted tasks that prioritize communicative agency, allowing learners to exercise control beyond rigid, prescribed prompts. For example, in a beginner Spanish unit on daily routines or personal preferences, students could first rehearse core expressions in class, then interact with AI through guided prompts, and finally extend the conversation by asking follow-up questions or introducing personally meaningful details. Similarly, developers may consider creating interactional formats that emphasize autonomy and discovery. Second, the delayed emergence of emotional benefits underscores the necessity of sustained and structured AI integration. LOTE instruction should move beyond one-off practice sessions and instead integrate AI-assisted speaking tasks over extended periods, emphasizing autonomy, exploration, and communicative agency. During the initial calibration period, teachers may need to scaffold learners’ use of AI by modelling prompt construction, demonstrating how to repair communication breakdowns, and helping students interpret AI feedback without treating it as formal evaluation. Given learners’ concerns about diminishing novelty and limited emotional authenticity, sustained implementation should also connect AI rehearsal with subsequent peer- or teacher-led communication. In this sense, AI-mediated practice may be most sustainable when used as a complementary rehearsal tool rather than as a replacement for human interaction. Third, the observed relationship between FLE and WTC suggests that cultivating positive affect may be a promising pedagogical pathway for promoting communicative willingness, complementing approaches that focus primarily on anxiety reduction. To achieve this, educators can combine structured classroom guidance with accessible AI-mediated interaction to cultivate learners who are more confident and engaged. Finally, the Spanish LOTE context reveals that while these learners may share certain gradual affective patterns with English learners, they face unique vulnerabilities such as limited authentic exposure. This indicates that AI-mediated speaking tools may be particularly valuable for LOTE education, providing accessible, emotionally supportive, and flexible interactional environments that can help bridge the gap between classroom study and authentic oral production.

Although this study provides valuable insights into AI-mediated speaking practice for beginner Spanish learners, several limitations warrant consideration and suggest directions for future research.

First, despite the longitudinal mixed-methods design, the study was conducted within a single semester (16 weeks), which may limit the observation of longer-term emotional and communicative development. While the repeated measures captured a delayed reduction in FLB, endpoint evidence for WTC, and a tentative upward tendency in FLE, it is unclear whether these patterns persist beyond the intervention or how sustained AI-mediated practice might influence advanced speaking proficiency. Moreover, given the modest sample size and the small FLA planned-contrast estimates relative to their standard errors, smaller changes in FLA may have been difficult to detect reliably. Future research could employ longer longitudinal designs, such as one academic year or two academic years, as well as follow-up assessments to track the durability of affective and communicative outcomes over time.

Second, the study primarily relied on self-report questionnaires and interview data, which may be subject to social desirability and common method bias. To assess the potential influence of common method bias in the quantitative data, Harman’s single-factor test was conducted. The first unrotated factor accounted for 35.25, 42.56, and 48.78% of the total variance at T1, T2, and T3, respectively. Although all values were below the commonly used 50% threshold, the T3 value was relatively close to this threshold; therefore, common method bias cannot be fully ruled out. While helped triangulate the quantitative findings, incorporating additional behavioral or performance-based measures, such as oral complexity, accuracy, fluency, pronunciation quality, interactional turn-taking, and task-completion quality in authentic conversational tasks, would strengthen the validity of conclusions and provide a more comprehensive understanding of learners’ communicative engagement. Relatedly, although the same construct domains, item stems, response format, and scoring procedures were retained across groups, the activity referents differed between the EG and the CG: EG items referred to in-class Spanish speaking activities that included AI-mediated tasks, whereas CG items referred to conventional classroom speaking activities. This parallel adaptation was intended to maintain ecological validity, but it may have introduced potential construct nonequivalence. Future studies should consider measurement invariance testing or parallel items that distinguish general classroom speaking emotions from AI-specific responses.

Third, because participation was voluntary, the sample may not fully represent the broader population of beginner Spanish learners. Students who consented to participate may have been more receptive to technology-enhanced learning or more willing to engage in speaking activities. In addition, group assignment was based on intact classes rather than individual randomization; therefore, unmeasured class-level differences cannot be fully ruled out despite the baseline comparisons. This potential self-selection and class-level effects should be considered when generalizing the findings to learners with lower initial motivation, less interest in AI-supported practice, or greater hesitation toward oral communication, as well as to other LOTE contexts with different learner profiles, course structures, and exposure opportunities.

Fourth, although the experimental group completed the required TalkPal AI tasks during monitored classroom segments, and students completed standardized tasks, and the instructor checked task completion through in-class observation, the present study did not include objective app-based usage logs to verify individual-level usage duration, interaction quality, or any voluntary out-of-class use beyond the structured classroom tasks. Future research should collect app-based usage logs, weekly self-reported usage records, or more systematic teacher-verified task-completion data to examine whether the intensity and quality of AI engagement moderate learning outcomes. Finally, while TalkPal AI offered a supportive, low-pressure environment, the AI’s responses lacked emotional nuance and full human authenticity. This limitation may constrain learners’ preparation for high-stakes or real-world communicative scenarios. Future research could explore more sophisticated AI designs that integrate adaptive feedback, multimodal cues, or culturally contextualized conversational content, as well as investigate the comparative efficacy of AI-mediated versus human-mediated practice for fostering resilient, authentic communicative competence.

## 6 Conclusion

In the context of LOTE education, supporting beginner learners’ emotional and communicative development is a key challenge for higher education. This study, through a mixed-methods longitudinal design involving 69 Chinese university students learning Spanish, suggests that AI-mediated speaking practice can foster meaningful affective and communicative changes over time. Quantitative data revealed that repeated AI interaction was most clearly associated with reduced foreign language boredom and higher willingness to communicate at the final measurement point, while foreign language enjoyment showed a positive but non-significant tendency and foreign language anxiety remained statistically stable. Qualitative interviews clarified that these changes emerged gradually as learners became more familiar with AI-mediated interaction and overcame initial cognitive and procedural hurdles.

The findings indicate that AI may provide a low-pressure, responsive, and supportive environment that gradually enhances learners’ sense of control and communicative agency. In the experimental group, increases in FLE were associated with increases in WTC over the full intervention period, suggesting that enjoyment may be closely linked to learners’ developing communicative readiness. This pattern highlights the cumulative and temporal nature of affective change in beginner LOTE learners. At the same time, stable foreign language anxiety underscores the persistent influence of evaluative pressures in high-stakes educational contexts. The qualitative findings further suggest that although AI can serve as a psychologically safe rehearsal space, its limited emotional authenticity means that it should complement rather than replace human-mediated communication.

In conclusion, these findings suggest that by integrating AI-assisted speaking tasks over extended periods, educators can cultivate learners who are more capable of bridging the gap between classroom learning and authentic oral production. Ultimately, this research underscores the potential of AI-mediated interaction to foster sustained affective and communicative development, providing a promising pathway for beginner learners to meet real-world communicative challenges.

## Data Availability

The raw data supporting the conclusions of this article will be made available by the authors, without undue reservation.

## Appendix A: interview questions

1. How do you feel about communicating in Spanish with AI chatbots?
2. How might AI chatbots have affected your WTC in Spanish?
3. Did you encounter challenges interacting with AI chatbots? If yes, please explain it.
4. What do you think are the differences between communicating with AI chatbots and communicating with humans? Which do you prefer?

## Appendix B: thematic codes generated from participants’ interview responses

**Table:**

Theme	Subtheme	Illustrative codes	Participants mentioning this subtheme
Psychologically safe rehearsal space	Reduced evaluative pressure	Low pressure; reduced peer or teacher judgment; reduced fear of mistakes	EG-2, EG-3, EG-4, EG-6, EG-8
	Non-threatening feedback and retry opportunities	Grammar verification; implicit correction; encouragement to continue; retrying without embarrassment	EG-3, EG-8
Early calibration difficulties	Linguistic resource constraints	Limited vocabulary; difficulty understanding AI replies; insufficient sentence resources	EG-4, EG-5, EG-6, EG-7
	Interaction management difficulties	Difficulty sustaining turns; prompt uncertainty; task-completion orientation; short responses	EG-1, EG-5, EG-7, EG-8
Growing communicative control and agency	Increasing interactional familiarity	Smoother interaction; getting used to expressing oneself; increased confidence; gradual habituation	EG-1, EG-2, EG-5, EG-7
	Expanding self-expression	Topic exploration; moving beyond assigned tasks; trying more complex sentences; using newly learned vocabulary	EG-1, EG-3, EG-5, EG-9
Persistent evaluation-related anxiety	Formal assessment pressure	Oral exam worry; teacher evaluation; exam-related anxiety	EG-06
	Public performance concern	Fear of embarrassment; public correction; concern about classmates’ reactions	EG-04, EG-08
Perceived limits of AI-mediated interaction	Diminishing novelty and predictability	Novelty fading; predictable responses; mechanical or task-oriented interaction	EG-01, EG-03, EG-09
	Limited emotional authenticity	Lack of emotional reciprocity; preference for human connection; AI as rehearsal rather than replacement	EG-01, EG-03, EG-09
